# Doxorubicin and folic acid-loaded zinc oxide nanoparticles-based combined anti-tumor and anti-inflammatory approach for enhanced anti-cancer therapy

**DOI:** 10.1186/s12885-023-11714-4

**Published:** 2024-01-04

**Authors:** Soha Gomaa, Mohamed Nassef, Ghada Tabl, Somia Zaki, Asmaa Abdel-Ghany

**Affiliations:** Zoology department, Faculty of Science, Tanta University, Tanta, 31527 Egypt

**Keywords:** ZnONPs, EAC, Anti-proliferative, Anti-tumor, Anti-inflammatory, Apoptosis

## Abstract

**Background:**

Zinc oxide nanoparticles (ZnONPs) have impressively shown their efficacy in targeting and therapy of cancer. The present research was designated to investigate the potential of ZnONP nanocomposites as a cancer chemotherapeutic-based drug delivery system and to assess the anti-tumor and anti-inflammatory effectiveness of ZnONP nanocomposites combination with systemic chemotherapeutic drugs doxorubicin (DOX) and folic acid (FA) in Ehrlich ascites carcinoma (EAC) tumor cell line both in vitro and in vivo.

**Methods:**

Anti-tumor potential of ZnONP nanocomposites: ZnONPs, ZnONPs/FA, ZnONPs/DOX and ZnONPs/DOX/FA against EAC tumor cell line was evaluated in vitro by MTT assay. Anti-tumor and anti-inflammatory efficacy of ZnONP nanocomposites were analyzed in vivo by examination of the proliferation rate and apoptosis rate of EAC tumor cells by flow cytometry, splenocytes count, level of inflammatory markers interleukin 6 (IL-6) and tumor necrosis factor alpha (TNF-α), as well as liver and kidney function in EAC-challenged mice.

**Results:**

In vitro results showed that ZnONP nanocomposites showed a high anti-proliferative potency against EAC tumor cells. Furthermore, the in vivo study revealed that the treatment EAC-challenged mice with ZnONPs, ZnONPs/DOX, ZnONPs/FA and ZnONPs/DOX/FA hindered the proliferation rate of implanted EAC tumor cells through lowering their number and increasing their apoptosis rate. Moreover, the treatment of EAC-challenged mice with ZnONPs/DOX/FA markedly decreased the level of IL-6 and TNF-α and remarkably ameliorated the liver and kidney damages that were elevated by implantation of EAC tumor cells, restoring the liver and kidney functions to be close to the naïve mice control.

**Conclusion:**

ZnONP nanocomposites may be useful as a cancer chemotherapeutic-based drug delivery system. ZnONP nanocomposites: ZnONPs/DOX, ZnONPs/FA and ZnONPs/DOX/FA regimen may have anti-inflammatory approaches and a great potential to increase anti-tumor effect of conventional chemotherapy, overcoming resistance to cancer systemic chemotherapeutics and reducing their side effects, offering a promising regimen for cancer therapy.

## Introduction

Cancer is one of the most common health problems, causing the largest number of deaths worldwide [1–3]. Doxorubicin (DOX) is a widely cancer chemotherapeutic used against tumors of diverse origins particularly breast cancer [4, 5]. A key mechanism by which DOX induces apoptosis in cancer cells involves its ability to intercalate within DNA base pairs, causing DNA strand breaks and inhibiting DNA and RNA synthesis, along with the inhibition of topoisomerase II [6]. It also induces the generation of reactive oxygen species (ROS) causing damage to cellular membranes, DNA and proteins [7]. Drug resistance and tumor growth are the most severe side effects for DOX, resulting in poor patient survival rates and poor prognosis [8, 9]. Its use is associated with severe adverse effects which impose a narrow therapeutic dose limiting DOX effectiveness [10, 11]. Despite significant progress regarding cancer treatments, therapeutic resistance and toxicity of conventional chemotherapeutics to normal tissues remain major concerns. Over 90% of patients with metastatic cancer fail their treatment because of chemo-resistance to chemotherapy drugs. In this regard, it is necessary to reduce DOX doses and modify its biodistribution in order to reduce its side effects and improve its concentration in tumors [12]. Among the various new ways to fight cancer, nanomedicine has attracted a lot of attention as it plays an important role in developing alternative and effective therapies for cancer treatment [13].

Nanoparticles (NPs)-based drug delivery system had been widely investigated for reducing the chemotherapeutic agents’ diverse side effects and improving their anti-tumor activity by specifically targeting the cancer cells [13–16]. NPs-based drug delivery system can take advantage of the unique disorganized vasculature of cancer cells with many pores and compromised lymphatic drainage to increase permeability and retention, which increases the accumulation of chemotherapeutic drugs in tumors and decrease their consumption by healthy tissues [17, 18].

Zinc oxide nanoparticles (ZnONPs) are among the most useful metal oxide nanoparticles in the treatment of cancer due to their excellent biocompatibility, low toxicity and capacity to specifically target and destroy cancer cells by selectively inducing ROS causing cancer cells apoptosis. ZnONPs are useful as a cancer chemotherapeutic NPs-based drug delivery system and have the capacity to target tumors specifically, giving them a potential alternative to traditional cancer treatments [16–19].

In addition to being able to cross the therapeutic indices like the other chemotherapeutic medicines ZnONPs may display strong cancer cell selectivity, retention, and controlled release of ligated as well as loaded therapies [20, 21]. ROS may provide a possible explanation for the selective cytotoxic response of ZnONPs towards proliferating cells. It has been observed that ROS generation is relatively greater in cancer cells than in normal cells after ZnONPs treatment. ROS and various signaling molecules are generally found in greater amount in rapidly proliferating cells such as cancer cells, owing to their faster metabolism rate compared with normal cells [22–24].

Numerous studies have demonstrated the selective cytotoxicity of ZnONPs towards cancer cells. However, the precise mechanism underlying this selectivity remains unclear. ROS may offer a reasonable explanation for the selective cytotoxic response of ZnONPs towards proliferating cells. It has been observed that cancer cells exhibit a relatively greater generation of ROS than normal cells following ZnONPs treatment [25–27]. Rapidly proliferating cells, such as cancer cells, typically contain higher levels of ROS and various signaling molecules due to their faster metabolism rate compared to normal cells [26]. Upon treatment with ZnONPs, the nanoparticles, being a redox reaction system in themselves, may react with the increased amount of chemical species and signaling molecules surrounding them, leading to the production of even more ROS. This results in significant oxidative stress in the cell, ultimately leading to cell death. While ZnONPs treatment also generates ROS in normal cells, the generation is relatively low compared to cancer cells, as initially they contain fewer ROS and signaling molecules that can be converted into more reactive species. Consequently, the oxidative stress produced may not be sufficient to induce cell death, resulting in a relatively lower cytotoxic response. Therefore, this could be the underlying mechanism for ZnONPs’ selective cytotoxicity in proliferating cells, such as cancer cells [28]. A collaborative approach could result in the development of intelligent NPs that are highly selective and toxic to cancer cells while not harming normal cells. In fact, this is a feasible goal, considering the promising properties of ZnONPs and their inherent selectivity and toxicity to cancer cells, which make them an essential tool for next generation cancer therapy [28].

For targeted drug delivery, folic acid (FA) molecules are conjugated to ZnONPs to target folate receptors, which are reported to be overexpressed on many cancer cells [29]. This tumor-targeting compound, FA, allows endocytosis of cancer cells and aggregates ZnONPs by recognizing its homolog, which is commonly expressed on the surface of many cancer cells [30, 31]. Therefore, labeling FA with ZnONPs may be a better medical strategy to target cancer cells as it offers high solubility, long-term diffusion and high biocompatibility in the produced nanomaterial [32, 33]. FA has a natural affinity towards folate receptor protein, which is over expressed by a number of tumor cells [34]. In addition to its targeted chemo-photothermal therapy synergistic effect, ZnONPs/DOX/FA transported heat and drug conspicuously to cancer cells. Consequently, the ZnONPs/DOX/FA system enhances targeted chemo-photothermal therapy and regulated drug release in a single system [29, 35].

ZnONP nanocomposites: ZnONPs/DOX, ZnONPs/FA and ZnONPs/DOX/FA were more effective in inhibiting the proliferation rate of EAC tumor cells compared to ZnONPs alone or DOX alone and could act as an effective drug delivery system for delivering DOX into EAC tumor cells and improving its chemotherapy effectiveness. The most rationale reason for this improved effectiveness of ZnONPs/DOX and ZnONPs/DOX/FA may be due to high drug loading efficacy that could markedly increase the intracellular penetration and hence concentration of DOX, thus improving the suppression growth of cancer cells. ZnONPs/DOX nanocomposite has an effective cytotoxic potential against breast cancer MCF-7 cells and colon cancer HT-29 cells comparing to ZnONPs alone or DOX alone [36]. The mechanism here may be due to that these ZnONP nanocomposites caused significant ROS generation, decreased mitochondrial potential and increased caspase-3 activation resulting in induction of mitochondria-mediated apoptosis in tumor cells [37]. In view of the above mentioned considerations, the current study was designated to investigate the potential of ZnONP nanocomposites as a cancer chemotherapeutic-based drug delivery system and to assess the anti-tumor and anti-inflammatory effectiveness of ZnONP nanocomposites in combination with systemic chemotherapeutic drug DOX and FA in Ehrlich ascites carcinoma (EAC) tumor cell line both in vitro and in vivo.

## Materials and methods

### Reagents

ZnONP nanocomposites were prepared in Nanotech lab (Inc., Cairo, Egypt). DOX was dissolved in phosphate buffer saline, PBS (Lonza, Bio Whittaker, USA) and frozen at -80 °C until use. FA used in this study was purchased from Sigma-Aldrich, USA. Roswell Park Memorial Institute medium 1640 (RPMI 1640) supplemented with heat-inactivated fetal bovine serum (FBS) (10% v/v), 2-mM L-glutamine and penicillin-streptomycin mixture (100 IU/ml), 1-mM sodium pyruvate, and non-essential amino acids (Invitrogen, USA). Ammonium chloride potassium (ACK), lysis buffer was purchased from Lonza (Bio Whittaker, USA). Tetrazolium MTT (3-(4, 5- dimethylthiazolyl-2)-2, 5-diphenyltetrazolium bromide) (ThermoFisher Scientific, USA) dissolved in 10% Dimethyl sulfoxide (DMSO) (Sigma, St. Louis, MO). Monoclonal antibodies: annexin V and PI were purchased from Pharmingen, San Diego, CA, USA.

### Synthesis of ZnONP nanocomposites

ZnONPs has been prepared according to the methods of Pacholski et al. [38], Beek et al. [39] and Seow et al. [40] through the hydrolysis and condensation of zinc acetate dihydrate by potassium hydroxide in alcoholic medium at low temperature condition. ZnONPs precipitated at the bottom. The excess mother liquor was removed and the precipitate was washed with methanol. The precipitate was then dispersed in a methanol mixture and chloroform. DOX-ZnONPs were prepared by coating DOX onto the outer surface of ZnONPs. ZnO nano powder dispersion (5 mg/mL) in distilled water (DW) and a DOX solution (5 mg/ml) in DW were mixed at a 1:1 volume ratio and incubated for 1 h at room temperature (RT). After centrifugation (8000 rpm, 15 min) the supernatant was removed and the NPs pellet was resuspended in DW. Finally, it freeze-dried for further uses. Amount of 0.2 g of reduced folate zinc oxide (rZnO/FA) was suspended in 100 ml of PBS (pH 7.4) then sonicated in ultrasonic water bath for1h at RT. 10 ml of DOX(50 mg/25 ml), (with observed UV spectrum at 480 nm, 2.822 cm-1) was added to rZnO/FA and stirred at dark condition overnight then centrifuged at 1500 rpm for 10 min at RT, After loading, UV spectrum of supernatant was observed at 480 nm was 0.460 cm-1. Synthesized ZnONP nanocomposites have been characterized by UV-Vis spectroscopy followed by transmission electron microscopy (TEM) to investigate the shape and size of prepared nanoparticles conjugates.

### In vitro anti-tumor activity assay of ZnONP nanocomposites

In vitro anti-tumor activity of ZnONP nanocomposites on EAC tumor cells was done using MTT assay. EAC tumor cell lines cultures were collected, washed three times and resuspended in PBS. Viable cells were counted using Trypan blue dye exclusion assay. EAC cells introduced to the RPMI-1640 medium at a concentration of 2 × 10^4^ cell/well in Corning^®^ 96-well tissue culture plates, which were then incubated for 24 h. The tested ZONP-nanocomposites were subsequently added to wells along with a reference drug DOX at concentrations of 10 µg/ml, 50 µg/ml and 100 µg/ml. Untreated controls with media or 0.5% DMSO were run for each 96 well plate as a control. After incubating for 72 h, the numbers of viable cells were determined by the MTT assay. Briefly, the media was withdrawn from each well and replaced with 100 µl of fresh culture media then 10 µl of the 12 mM MTT stock solution (5 mg of MTT in 1 mL of PBS) was added to each well. The 96 well plates were then incubated at 37 °C and 5% CO_2_ for 4 h. 50 µl of DMSO was added to each well and mixed thoroughly with pipetting. Then the plate was incubated at 37 °C for 10 min. The optical density was measured at 570 nm with the microplate reader (Bio-Rad microplate reader, Japan) to determine the cell viability and proliferative rate.

The percentage of viability was calculated according to the following equation:

$${\rm{\% }}\,{\rm{viability}}\,{\rm{ = }}\,\left( {{\rm{AT}}\,{\rm{-}}\,{\rm{AB}}} \right)\,{\rm{/}}\,\left( {{\rm{AC}}\,{\rm{-}}\,{\rm{AB}}} \right)\,{\rm{ \times }}\,{\rm{100}}$$

Where, AT is the absorbance of treated cells (drug), AB is the absorbance of blank (only media) and AC is the absorbance of control (untreated).

The 50% inhibitory concentration (IC_50_) values for the EAC tumor cell line after 72 h were estimated using graphpad prism software (San Diego, CA, USA) at 6.5 µg/ml, 10.8 µg/ml, 20.6 µg/ml, 8.3 µg/ml and 38.8 µg/ml for DOX, ZnONPs, ZnONPs/DOX, ZnONPs/FA and ZnONPs/DOX/FA, respectively (Table 1). In vivo 50% lethal dose (LD_50_) values, calculated from the IC_50_ values according to the formula: log (LD_50_) = 0.372 × log (IC_50_) + 2.024 [44], estimated at 212 mg/kg (5.3 mg/mouse), 256 mg/kg (6.4 mg/mouse), 326 mg/kg (8.2 mg/mouse), 232 mg/kg (5.8 mg/mouse), and 412 mg/kg (10.3 mg/mouse) for DOX, ZnONPs, ZnONPs/DOX, ZnONPs/FA, and ZnONPs/DOX/FA, respectively. the sublethal doses of 1/13 of LD_50_ value of each ZnONPs nanocomposite were chosen for in vivo intraperitoneal (IP) treatment at 0.4 mg/mouse, 0.5 mg/mouse, 0.6 mg/mouse, 0.4 mg/mouse, and 0.8 mg/mouse for DOX, ZnONPs, ZnONPs/DOX, ZnONPs/FA, and ZnONPs/DOX/FA, respectively.

**Table 1:** In vitro growth inhibition concentration (IC50) (µg/ml) and In vivo estimated LD50 (mg/kg) of ZnONP nanocomposites on EAC tumor cell line

Nanocomposites	In vitro IC_50_ (µg/ml)	In vivo LD_50_ (mg/kg)
DOX	6.5	212
ZnONPs	10.8	256
ZnONPs/DOX	20.6	326
ZnONPs/FA	8.3	232
ZnONPs/DOX/FA	38.8	412

### Mice

Seventy female Swiss albino mice (6–8 weeks old, weighing 25 ± 2 g) were purchased from National Research Centre Animal House (Dokki, Giza, Egypt). Mice were distributed into 7 groups (n = 10) and given a standard pellet diet and tap water *ad libitum*. The experimental protocol was carried out following the guidelines for the Institutional Animal Care and Use Committee (IACUC) set forth by Faculty of Science, Tanta University, Tanta, Egypt (Approval Number: IACUC-SCI-TU-0062) regarding animal care, housing, and procedures minimize the suffering and distress of animals. There was strict adherence to the ARRIVE guidelines throughout all of the procedures carried out during the research to ensure that the animals were receiving the best possible care during the process. Moreover, all procedures are conducted ethically and humanely at all times.

### Tumor cell line and Tumor model preparation

EAC tumor cell line (Pharmacology and Experimental Oncology Unit, National Cancer Institute, Cairo University, Cairo, Egypt) was maintained in ascitic form in naïve female Swiss albino mice by weekly IP inoculation of 1 × 10^6^ cells/mouse as described in Gothoskar and Ranadive [42] and Abdel Salam et al. [43] The ascitic fluid, containing EAC cells, was gathered and resuspended in PBS, and the EAC cells were counted using Trypan Blue dye exclusion assay in a Neubauer hemocytometer. To prepare the tumor model, 2.5 × 10^5^ EAC cells were implanted through IP injection into naïve female Swiss albino mice.

### Tumor challenge and in vivo study design

Sixty female Swiss albino mice were IP injected with 2.5 × 10^5^ EAC cells/mouse then they divided randomly into 6 groups (n = 10). On day 7 post EAC challenged, group 1 (EAC group) was IP administered with PBS, group 2 (DOX group) was IP injected with DOX (0.4 mg/mouse), group 3 (ZnONPs group) IP received ZnONPs (0.5 mg/mouse), group 4 (ZnONPs/DOX group) was IP inoculated with ZnONPs/DOX (0.7 mg/mouse), group 5 (ZnONPs/FA group) was IP administrated with ZnONPs/FA (0.5 mg/mouse), and group 6 (ZnONPs/DOX/FA group) was IP administrated with ZnONPs/DOX/FA (0.8 mg/mouse) once/day for seven days. In addition to naïve mice group (naïve group), with ten mice, was IP administrated PBS. On day 15 post EAC inoculations, all groups of mice were anesthetized using Isoflurane, sacrificed, EAC tumor cells and spleen were harvested, sera were collected for biochemical analysis and the anti-tumor and the anti-inflammatory efficacy were assessed.

### Tumor cells harvesting and counting

Ascitic EAC tumor cells were collected individually from EAC-treated and -EAC-nontreated mice after being sacrificed by cervical dislocation. The EAC cells were resuspended in PBS and washed twice. Erythrocytes were lysed with ACK. EAC cells suspensions were centrifuged (3000 rpm, 5 min, 4 °C). The EAC cell pellets washed and resuspended in PBS. EAC cells count and viability were investigated by a Trypan blue dye exclusion assay.

### Splenocytes harvesting and counting

Splenocytes single-cell suspension of spleen were prepared as described previously by Nassef [44] and Gomaa [45]. Briefly, mice were sacrificed and spleens were aseptically removed and placed individually in a 60 mm × 15 mm petri dish with PBS. Splenocytes were isolated by dissociating spleen on 60 μm mesh sieves screens (Sigma, St. Louis, MO) and lysing of RBC was carried out with ACK buffer. Splenocytes were washed, counted and diluted in RPMI 1640 provided with 5% fetal calf serum (FCS) for further investigations.

### Assessment of apoptosis by flow cytometry

Ascitic EAC cells, collected from treated and untreated EAC-challenged were washed by ice-cold PBS, the cell density was calculated, and the EAC cells were resuspended in 1X annexin-binding buffer to obtain a final density of 1 × 10^6^ cells/ml. 100 µL of the cell suspension was placed into 1.5-ml eppendorf tubes and 5-µL annexin V-fluoresceinisothiocyanate (FITC), and 1 µL PI (100 µg/ml) working solution was added. After incubating stained EAC cells at room temperature for 15 min, 400 µL of 1X annexin-binding buffer was added, gently mixed, and the samples were then stored on ice. The cells were subsequently subjected to a flow cytometric analysis.

### Assay of inflammatory cytokines

The assessment of serum levels of inflammatory cytokines IL-6 and TNF-α was assessed by the enzyme-linked immunosorbent assay (ELISA) method with a commercially available kit (Rockford, Ill; Fisher Thermo Scientific Co, USA), in accordance with the manufacturer’s instructions. Briefly, one day prior to running the assay, 96-well plates were coated with the capture antibody. Following 18 h incubation at 4 °C, the plates were washed with PBS containing 0.05% Tween-20 (Sigma-Aldrich, St. Louis, MO, USA) and then incubated for 1 h at room temperature (RT) with a diluent buffer to block nonspecific binding. Following a wash, 100 µl of sample (100 g) was added to each well, and they were left to incubate for 2 h at room temperature. After washing of the plates, 100 µl biotinylated detection antibody was transferred to each well following by plates’ incubation for 1 h. Following this, 100 µl avidin-horseradish peroxidase (HRP) was added to each well followed by incubation for 30 min at room temperature. After further washing, 3,3′,5,5′-tetramethylbenzidine (TMB) substrate solution was added and the plates were incubated in the dark for 15 min. 100 ml of 2 N sulfuric acid was added to stop the reaction and the absorbance at 450 and 570 nm was measured.

### Analysis of liver and kidney functions

The levels of serum aspartate aminotransferase (AST) (U/l), alanine aminotransferase (ALT) (U/l)), creatinine (mg/dl) and urea (mg/dl) were colorimetrically evaluated by a fully-automatic biochemical analyzer (Vita lab Selectra E, German) using the standard available commercial kit (BIOLABO SAS, Les Hautes Rives, 02160, Maizy, France). The manufacturer’s manuals were precisely followed throughout the experiment.

### Statistical analysis

Data were represented as mean ± standard deviation (SD). Results were analyzed by one-way analysis of variance (ANOVA) followed by *post hoc* Tukey HSD’s test and Dennett’s test. *P* < 0.05 was considered significant.

## Results

### Characterization of ZnONP nanocomposites

**Fig. 1:**
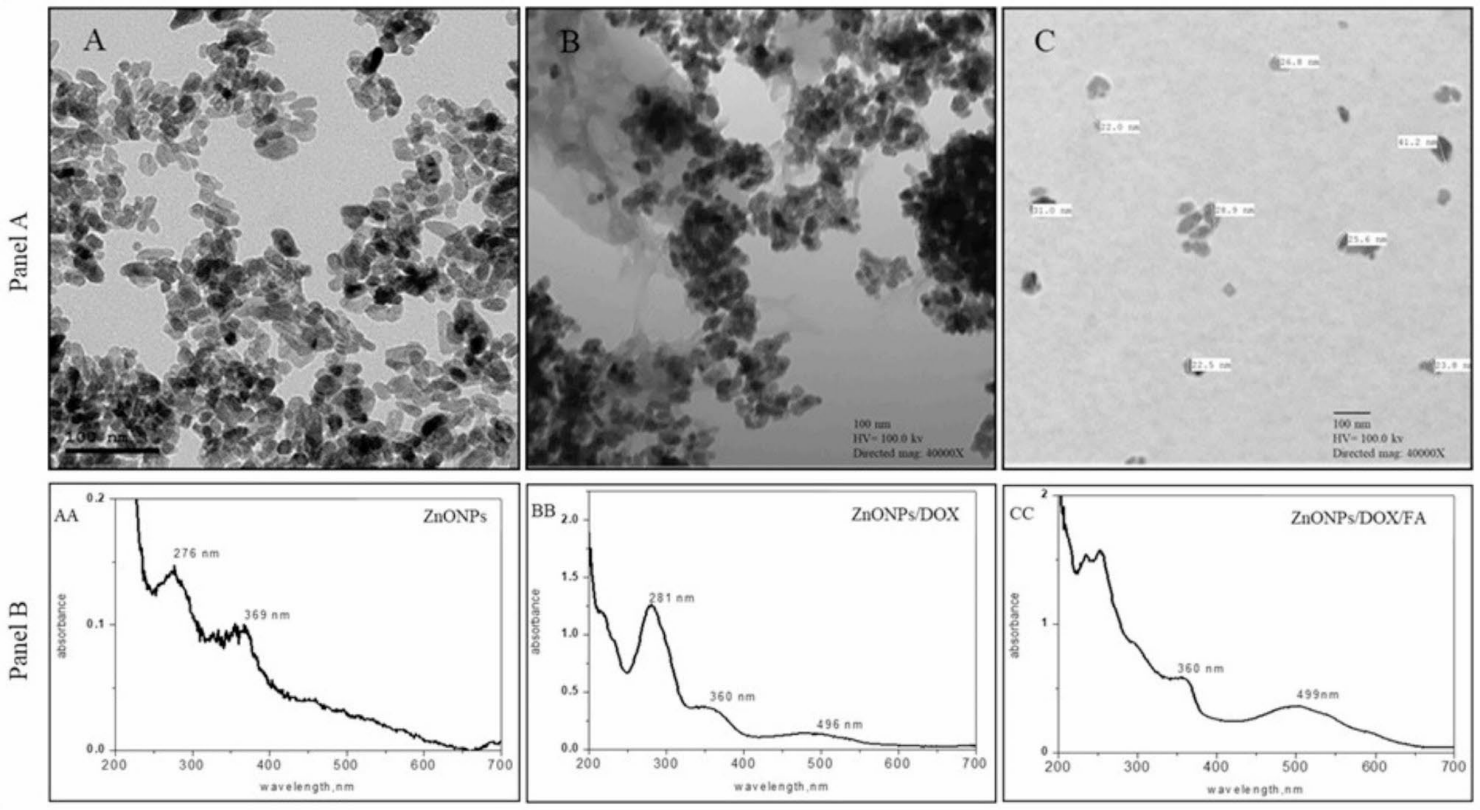
Characterization of ZnONP nanocomposites by transmission electron microscopy (TEM) (panel 1) and UV-Vis characterization spectra of ZnONP nanocomposites (panel 2). (**A**) ZnONPs, (**B**) ZnONPs loaded with DOX (ZnONPs/DOX), (**C**) ZnONPs loaded with DOX and FA (ZnONPs/DOX/FA), (AA) ZnONPs, (BB) ZnONPs loaded with DOX (ZnONPs/DOX) and (CC) ZnONPs loaded with DOX and FA (ZnONPs/DOX/FA)

The size and the shape of ZnONP nanocomposites were analyzed using TEM. The total ZnONPs size in the current study was approximately 20 nm expressing irregular arranged semispherical particles and agglomerated morphology (Fig. 1A). The morphology and size of the ZnONPs loaded with DOX (ZnONPs/DOX) appeared spherical structure with approximate size of 19–23 nm (Fig. 1B). However, ZnONPs loaded with DOX and FA (ZnONPs/DOX/FA) appeared spherical structure with approximate size of 22–41 nm (Fig. 1C). Further, UV-Visible characterization of free ZnONPs, ZnONPs/DOX and ZnONPs/DOX/FA were analyzed via UV–vis Spectrophotometer (Shimadzu, UV-2450). Synthesized ZnONPs absorption spectra formed in the reaction mixture were detected at 276 and 369 nm corresponded to the nanosized ZnO (Fig. 1AA). By loading DOX to the synthesized ZnONPs, the shifted peak appeared at 281 and 360 nm, which was related to loading of DOX onto ZnONPs (Fig. 1BB). Additionally, loading of FA onto ZnONPs/DOX composite showed peak at 360 and 499 nm (Fig. 1CC).

### In vitro anti-tumor activity assay

**Fig. 2:**
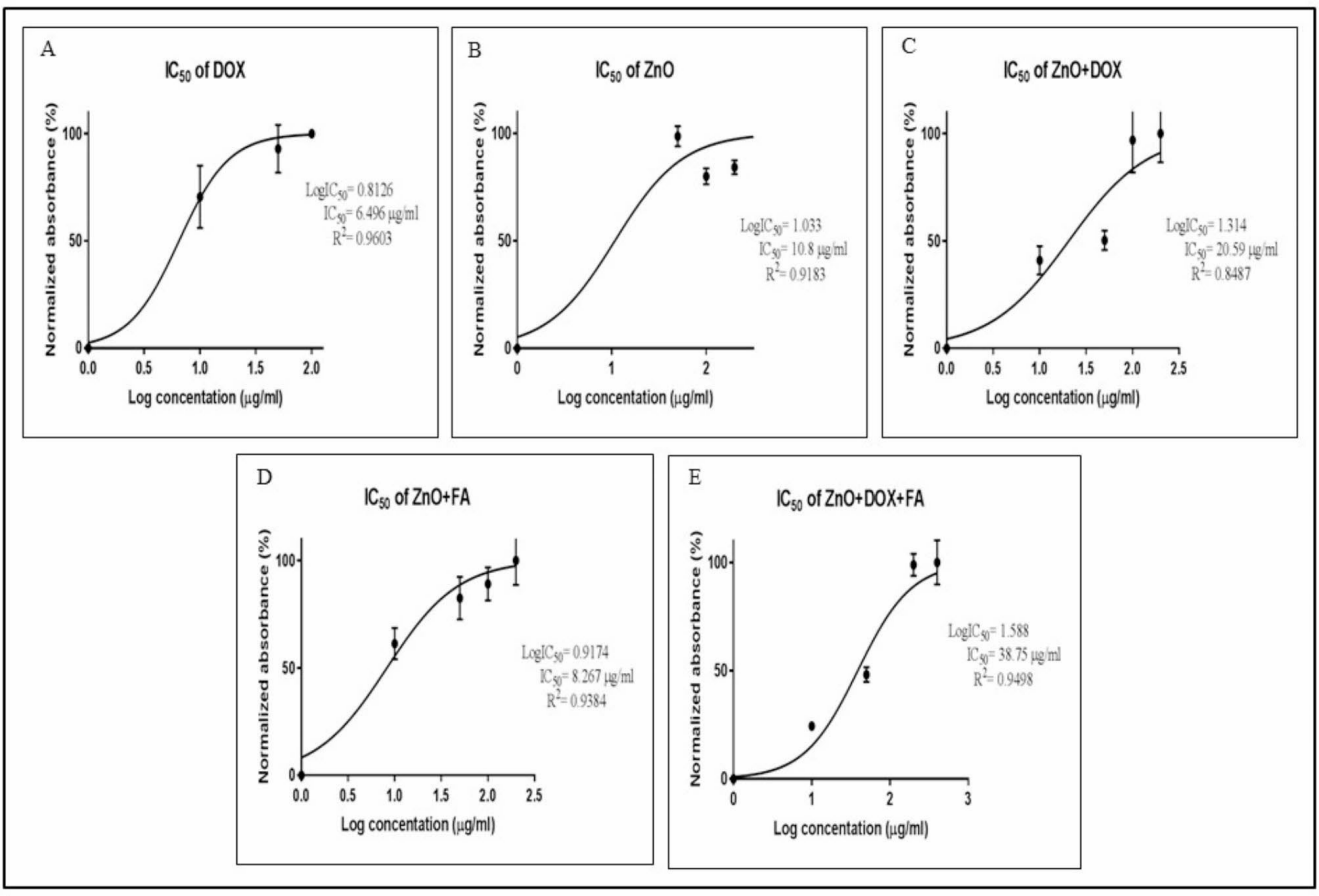
Effects of ZnONP nanocomposites on growth inhibition rate of EAC tumor cell line after 72 h of In vitro treatment using MTT assay. (**A**) DOX, (**B**) ZnONPs, (**C**) ZnONPs loaded with DOX (ZnONPs/DOX). (**D**) ZnONPs loaded with FA (ZnONPs/FA). (**E**) ZnONPs loaded with DOX and FA (ZnONPs/DOX/FA)

The results of the in vitro anti-tumor activity assay of ZnONP nanocomposites indicated that the treatment of EAC tumor cells with ZnONPs, ZnONPs/FA, ZnONPs/DOX and ZnONPs/DOX/FA increased the growth inhibition rate of EAC cells in dose dependent manners comparing to that in DOX-treated EAC cells (Fig. 2A-E). The estimated in vitro IC_50_ values of ZnONP nanocomposites on the EAC tumor cell line at 72 h post- ZnONP nanocomposites treatment were estimated at 6.5 µg/ml, 10.8 µg/ml, 20.6 µg/ml, 8.3 µg/ml and 38.8 µg/ml for DOX, ZnONPs, ZnONPs/DOX, ZnONPs/FA and ZnONPs/DOX/FA, respectively (Fig. 2A-E; Table 1). The in vivo LD_50_ values were estimated from the in vitro IC_50_ values at 212 mg/kg (5.3 mg/mouse), 256 mg/kg (6.4 mg/mouse), 326 mg/kg (8.2 mg/mouse), 232 mg/kg (5.8 mg/mouse), and 412 mg/kg (10.3 mg/mouse) for DOX, ZnONPs, ZnONPs/FA, ZnONPs/DOX and ZnONPs/DOX/FA, respectively (Table 1). The sublethal doses of 1/13 of LD_50_ value of each ZnONP nanocomposite were chosen for in vivo IP treatment at 0.4 mg/mouse, 0.5 mg/mouse, 0.6 mg/mouse, 0.4 mg/mouse, and 0.8 mg/mouse for DOX, ZnONPs, ZnONPs/DOX, ZnONPs/FA, and ZnONPs/DOX/FA, respectively.

### In vivo anti-tumor activity of ZnONP nanocomposites

**Fig. 3:**
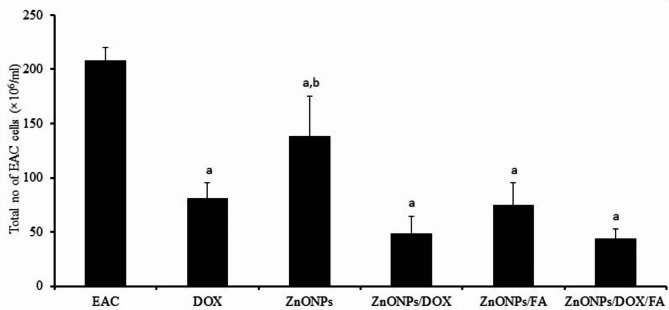
In vivo anti-tumor activity of ZnONP nanocomposites on EAC-challenged mice. EAC-challenged mice IP inoculated with PBS, DOX (0.4 mg), ZnONPs (0.5 mg), ZnONPs/DOX (0.7 mg), ZnONPs/FA (0.5 mg) or ZnONPs /DOX/FA (0.8 mg). Mice were sacrificed on day 11 post tumor challenge and Ascitic EAC tumor cells were harvested to determine their viability using trypan blue viability test. Data were represented as mean ± SD (n = 10). Difference between groups was considered statistically significant at *P* < 0.05. Note: ^a,b^ Statistically significant difference as compared to the corresponding means of the EAC group (**a**), the DOX group (**b**) within each column

Our findings demonstrated that the administration of DOX, ZnONPs, ZnONPs/DOX, ZnONPs/FA, and ZnONPs/DOX/FA to EAC-challenged mice resulted in a significant reduction in the number of EAC tumor cells (81.26 × 10^6^, 138.26 × 10^6^, 48.40 × 10^6^, 74.66 × 10^6^, 43.60 × 10^6^, respectively) compared to those mice that received only PBS (208.00 × 10^6^) (Fig. 3). Furthermore, treatment with ZnONPs led to a significant increase in the total number of tumor cells (138.26 × 106), while treatment with ZnONPs/DOX, ZnONPs/FA, and ZnONPs/DOX/FA resulted in an insignificant decrease in the count of tumor cells (48.40 × 10^6^, 74.66 × 10^6^, 43.60 × 10^6^, respectively) compared to those mice that received DOX (81.26 × 10^6^) (Fig. 3).

### Splenocytes harvesting and counting

**Fig. 4:**
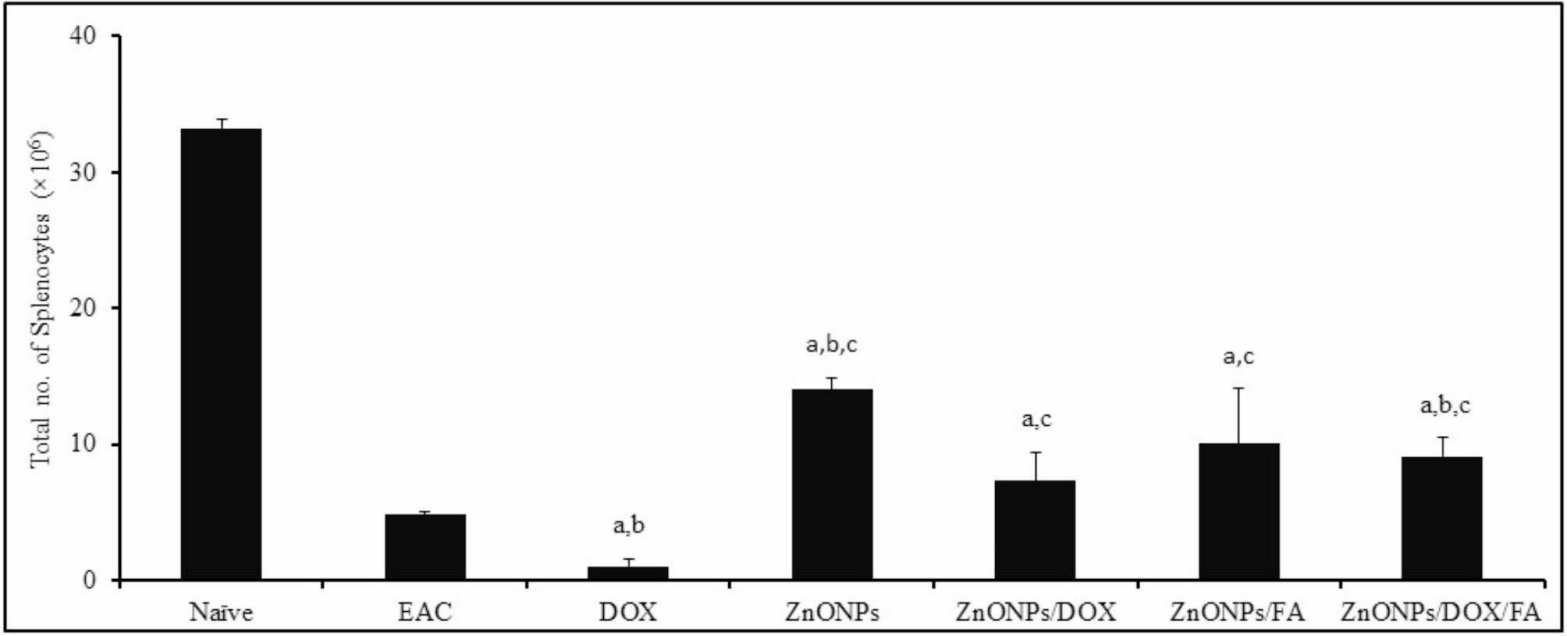
Potentials of ZnONPs conjugates on the total number of splenocytes in EAC-bearing mice. EAC-bearing mice IP inoculated with EAC-challenged mice IP inoculated with PBS, DOX (0.4 mg), ZnONPs (0.5 mg), ZnONPs/DOX (0.7 mg), ZnONPs/FA (0.5 mg) or ZnONPs /DOX/FA (0.8 mg). Mice were sacrificed on day 11 post tumor implantation and splenocytes were harvested to determine their viability and their total number using trypan blue viability test. Data were represented as mean ± SD (n = 10). Difference between groups was considered statistically significant at *P* < 0.05. Note: ^a,b,c^ Statistically significant difference as compared to the corresponding means of the naive group (**a**), the EAC group (**b**), the DOX group (**c**) within each column

Additionally, our findings demonstrate that the administration of various nanocomposites, including DOX, ZnONPs, ZnONPs/DOX, ZnONPs/FA, or ZnONPs/DOX/FA, to EAC-challenged mice resulted in a significant decrease in the total count of splenocytes (1.06 × 106, 14.05 × 106, 7.30 × 106, 10.03 × 106, and 9.12 × 106, respectively) compared to naïve mice that received PBS (33.26 × 106) (Fig. 4). Interestingly, treatment with ZnONPs or ZnONPs/DOX/FA significantly increased the count of splenocytes (14.05 × 106 and 9.12 × 106, respectively), while DOX treatment significantly reduced the total number of splenocytes (1.06 × 106) compared to EAC-challenged mice who received PBS (4.86 × 106) (Fig. 4). Furthermore, treatment with ZnONPs, ZnONPs/DOX, ZnONPs/FA, or ZnONPs/DOX/FA resulted in a significant increase in the count of splenocytes (14.05 × 106, 7.30 × 106, 10.03 × 106, and 9.12 × 106, respectively) comparing to that in EAC-challenged mice received DOX (1.06 × 106) (Fig. 4).

### Apoptosis assessment by flow cytometry

**Fig. 5:**
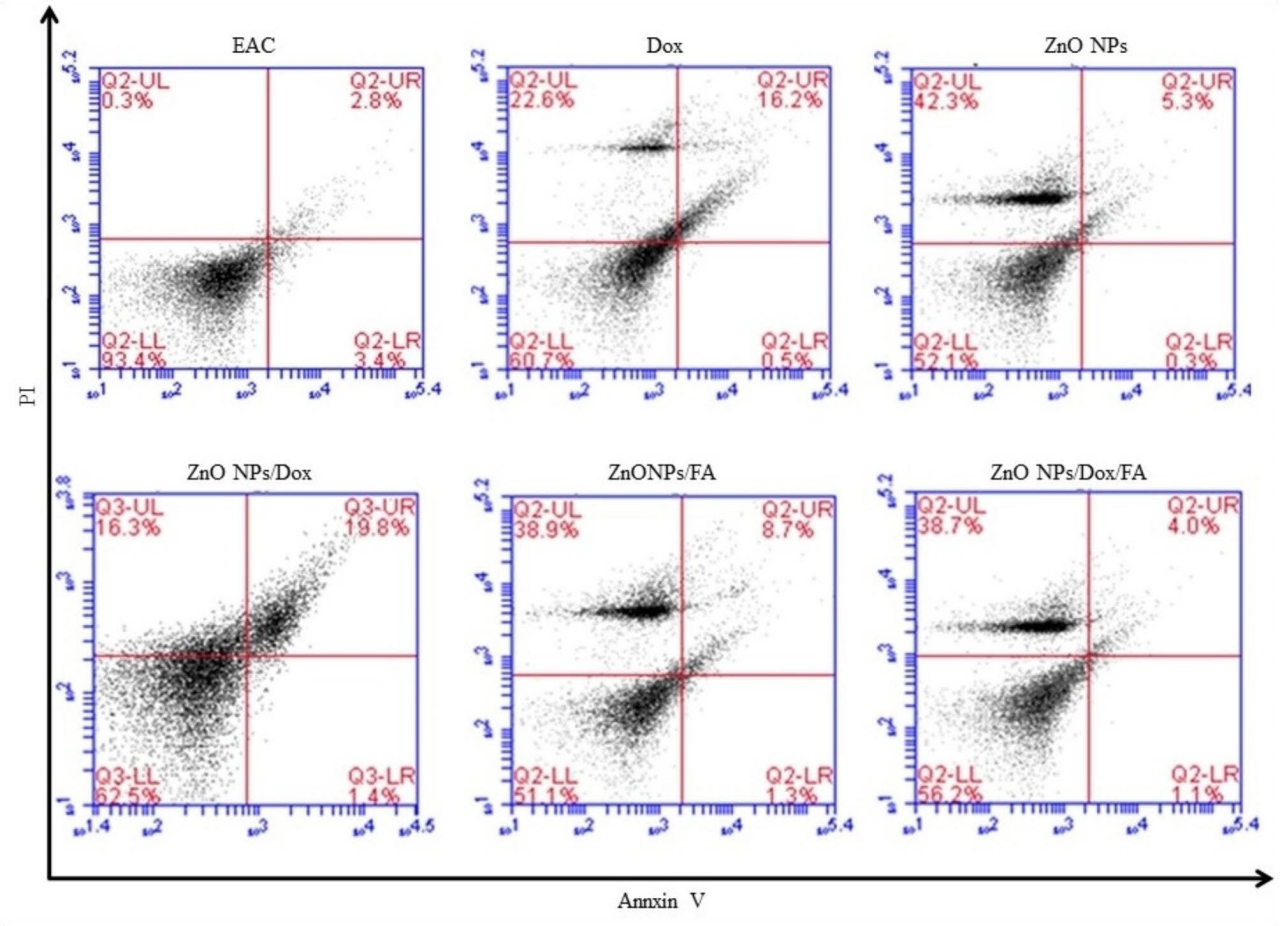
Phenotypic analysis of EAC tumor cells in EAC-challenged mice treated with ZnONP nanocompostes. EAC-challenged mice IP inoculated with PBS, DOX (0.4 mg), ZnONPs (0.5 mg), ZnONPs/DOX (0.7 mg), ZnONPs/FA (0.5 mg) or ZnONPs /DOX/FA (0.8 mg). Ascitic EAC cells were harvested from peritoneal cavity and washed twice with PBS. Cells were stained with Propidium Iodide (PI) and annexin V then analyzed by flow cytometry for the marker indicated on the representative histograms

The current results showed that the administration of EAC-challenged mice with DOX, ZnONPs, ZnONPs/DOX, ZnONPs/FA or ZnONPs/DOX/FA nanocomposites resulted in increase in the percentage of necrosis (22.6%, 42.3%, 16.3%, 38.9% and 38.7%, respectively) comparing to that in EAC-challenged mice received PBS alone (0.3%) (Fig. 5). Additionally, administration of EAC-challenged mice with DOX, ZnONPs, ZnONPs/DOX and ZnONPs/FA ZnONPs/DOX/FA led to decrease in the early apoptosis rate of EAC tumor cells (0.5%, 0.3%, 1.4%, 1.3%, and 1.1%, respectively) comparing to that in EAC-challenged mice received PBS alone (3.4%) (Fig. 5). Furthermore, the injection of EAC-challenged mice with DOX, ZnONPs/DOX or ZnONPs/FA increased the late apoptosis percentage of EAC tumor cells (16.2%, 19.8%, 8.7%, respectively), however EAC-challenged mice treated with ZnONPs or ZnONPs/DOX/FA showed slight increase in the late apoptosis percentage of EAC tumor cells (5.3% and 4%, respectively) comparing to that in EAC-challenged mice received PBS alone (2.8%) (Fig. 5).

**Fig. 6:**
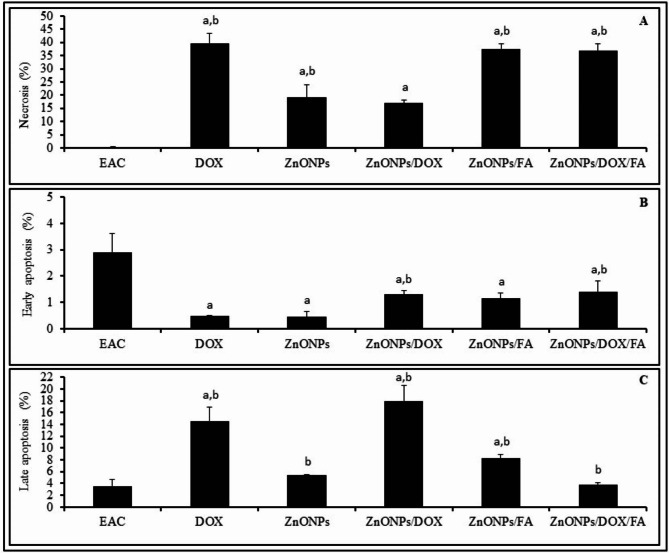
Potentials of of ZnONPs nanocomposites treatment on EAC cells in EAC-challenged mice treated with ZnONPs nanocomposites. EAC-challenged mice IP inoculated with PBS, DOX (0.4 mg), ZnONPs (0.5 mg), ZnONPs/DOX (0.7 mg), ZnONPs/FA (0.5 mg) or ZnONPs /DOX/FA (0.8 mg). Ascitic EAC cells were harvested from peritoneal cavity and washed twice with PBS. Data were represented as mean ± SD (n = 10). Difference between groups was considered statistically significant at *P* < 0.05. Note: ^a,b^ Statistically significant difference as compared to the corresponding means of EAC group (**b**), the DOX group (**c**) within each column

The findings here indicated that the necrosis % of ascitic EAC tumor cells significantly increased in EAC-challenged mice received DOX, ZnONPs, ZnONPs/DOX, ZnONPs/FA or ZnONPs/DOX/FA comparing to that in EAC-challenged mice received PBS (39.70%, 19.10%, 17.05%, 37.552% and 36.75%, respectively versus 0.30%). EAC-challenged mice IP injected with ZnONPs, ZnONPs/DOX, ZnONPs/FA or ZnONPs/DOX/FA recorded a significant decrease in the necrosis percentage of ascitic EAC tumor cells comparing to that in EAC-challenged mice received DOX (19.10%, 17.05%, 37.552% and 36.75%, respectively versus 39.70%) (Fig. 6A). The treatment of EAC-challenged mice with DOX, ZnONPs, ZnONPs/DOX, ZnONPs/FA and ZnONPs/DOX/FA significantly diminished the early apoptosis percentage of EAC tumor cells (0.50%, 0.45%, 1.30%, 1.15% and 1.40%, respectively) comparing to EAC-challenged mice received PBS alone (2.90%) (Fig. 6B). Additionally, the treatment of EAC-challenged mice with ZnONPs, ZnONPs/DOX, ZnONPs/FA and ZnONPs/DOX/FA significantly decreased the early apoptosis % of EAC tumor cells comparing to that in EAC-challenged mice received DOX (0.45%, 1.30%, 1.15% and 1.40%, respectively versus 0.50%) (Fig. 6B). The results in Fig. 6C show that the administration of EAC-challenged mice with DOX, ZnONPs/DOX or ZnONPs/FA nanocomposites resulted in a significant increase in the late apoptosis percentage comparing to that in EAC-challenged mice received PBS alone (14.60%, 5.3510%, 18.00%, 8.30% and 3.85%, correspondingly versus 3.55%). Interestingly, IP inoculation of EAC-challenged mice with ZnONPs/DOX significantly increased the late apoptosis % of EAC tumor cells, while their inoculation with ZnONPs/FA significantly decreased the late apoptosis % of EAC tumor cells comparing to that in EAC-challenged mice received DOX (14.60% and 18.00%, correspondingly versus 14.60%) (Fig. 6C).

### Inflammatory cytokines assay

**Fig. 7:**
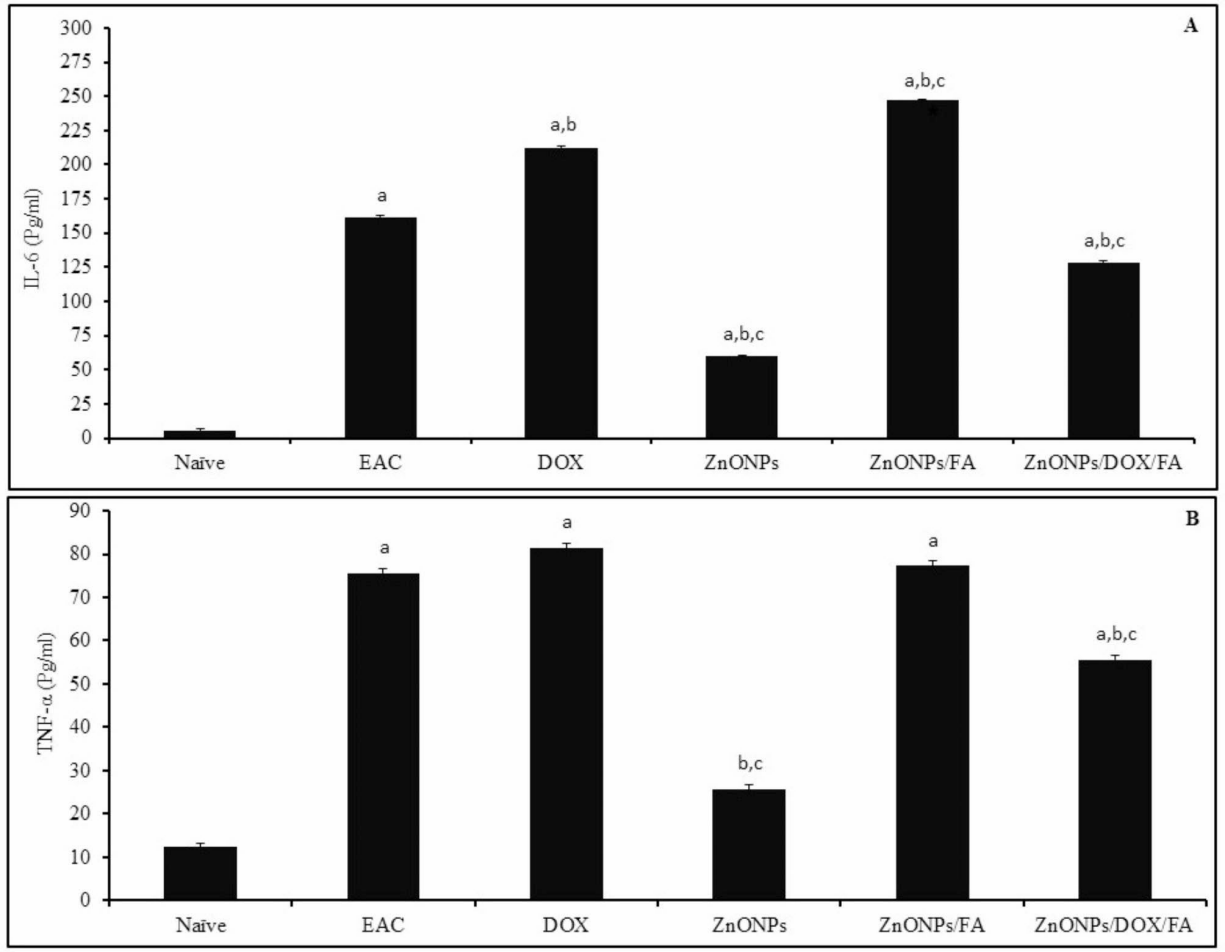
Potentials of ZnONP nanocomposites on the serum level of pro-inflammatory cytokines IL-6 (A) and TNF-α (B) in EAC-challenged mice. EAC-challenged mice IP inoculated with PBS, DOX (0.4 mg), ZnONPs (0.5 mg), ZnONPs/DOX (0.7 mg), ZnONPs/FA (0.5 mg) or ZnONPs /DOX/FA (0.8 mg). Mice were sacrificed on day 11 post tumor challenge and the sera samples were collected. Data were represented as mean ± SD (n = 10). Difference between groups was considered statistically significant at *P* < 0.05. Note: ^a,b,c^ Statistically significant difference as compared to the corresponding means of the naive group (**a**), the EAC group (**b**), the DOX group (**c**) within each column

The data here indicated that the level of IL-6 in the serum significantly increased in EAC-challenged mice received PBS, DOX, ZnONPs, ZnONPs/FA or ZnONPs/DOX/FA comparing to that in naïve mice (161 pg/ml, 212 pg/ml, 59 pg/ml, 247 pg/ml and 128 pg/ml, respectively versus 5 pg/ml) (Fig. 7A). EAC-challenged mice IP inoculated with DOX or ZnONPs/FA showed significant increase in IL-6 level (212 pg/ml and 247 pg/ml, respectively) and contrarily EAC-challenged mice IP injected with ZnONPs and ZnONPs/DOX/FA showed a significant decrease in IL-6 level (59 pg/ml and 128 pg/ml, respectively) comparing to that in EAC-challenged mice received PBS alone (161 pg/ml) (Fig. 7A). The treatment of EAC-challenged mice with ZnONPs or ZnONPs/DOX/FA significantly decreased the serum IL-6 level (59 pg/ml and 128 pg/ml, correspondingly), while their treatment with ZnONPs/FA significantly increased the IL-6 level in the serum (247 pg/ml) comparing to that in EAC-challenged mice received DOX (212 pg/ml) (Fig. 7A). Furthermore, our data revealed that TNF-α level significantly increased in EAC-challenged mice received PBS, DOX, ZnONPs/FA or ZnONPs/DOX/FA comparing to naïve mice (75 pg/ml, 81 pg/ml, 77 pg/ml and 55 pg/ml, respectively versus 12 pg/ml). EAC-challenged mice IP inoculated with ZnONPs and ZnONPs/DOX/FA revealed significant decrease in TNF-α level (25 pg/ml and 55 pg/ml, respectively) comparing to that in EAC-challenged mice received PBS alone (75 pg/ml) (Fig. 7B). Comparing to EAC-challenged mice injected with DOX, treatment of EAC-challenged mice with ZnONPs or ZnONPs/DOX/FA significantly decreased the serum level of TNF-α (81 pg/ml versus 25 pg/ml and 55 pg/ml, respectively) (Fig. 7B).

### Analysis of liver and kidney functions

Our experiments revealed that the serum level of ALT significantly increased in EAC-challenged mice received PBS, DOX, ZnONPs, ZnONPs/DOX, ZnONPs/FA or ZnONPs/DOX/FA comparing to that in naïve mice (78 U/L, 80 U/L, 47 U/L, 62 U/L, 59 U/L and 60 U/L, respectively versus 30 U/L) (Table 2). EAC-challenged mice IP injected with DOX, ZnONPs, ZnONPs/DOX, ZnONPs/FA or ZnONPs/DOX/FA revealed significant decrease in ALT level (80 U/L, 47 U/L, 62 U/L and 59 U/L and 60 U/L, respectively versus 78 U/L) (Table 2). The treatment of EAC-challenged mice with ZnONPs, ZnONPs/DOX, ZnONPs/FA or ZnONPs/DOX/FA significantly decreased the serum level of serum ALT (47 U/L, 62 U/L and 59 U/L and 60 U/L, respectively) comparing to that in EAC-challenged mice received DOX (80 U/L) (Table 2). Additionally, the data in Table 2 show that serum level of AST significantly increased in EAC-challenged mice received PBS, DOX, ZnONPs/DOX, ZnONPs/FA and ZnONPs/DOX/FA comparing to that in naïve mice (215 U/L, 240 ± U/L, 347 U/L, 244 U/L and 237 U/L, respectively versus 95 U/L). EAC-challenged mice IP treated with DOX or ZnONPs/DOX recorded a significant increase in serum AST level (240 U/L and 347 U/L, respectively) comparing to that in EAC-challenged mice received PBS alone (215 U/L) (Table 2). Comparing to EAC-challenged mice received DOX, IP injection of EAC-challenged mice with ZnONPs/DOX significantly increased the serum level of AST (240 U/L versus 347 U/L) (Table 2).

**Table 2:** Potentials of ZnONP nanocomposites on the liver function of EAC-challenged mice

Conjugates	ALT (U/L)	AST (U/L)
Naive	30.33 ± 5.50	95.67 ± 5.13
EAC	77.98 ± 0.57^a^	215.26 ± 9.39^a^
DOX	80.11 ± 5.55^a^	240.43 ± 8.97^a,b^
ZnONPs	47.47 ± 2.99^a,b,c^	298.13 ± 24.49
ZnONPs/DOX	62.26 ± 5.78^a,b,c^	347.60 ± 30.36^a,b,c^
ZnONPs/FA	58.94 ± 6.98^a,b,c^	244.76 ± 25.05^a^
ZnONPs/DOX/FA	60.20 ± 6.75^a,b,c^	237.87 ± 28.33^a^

Data were represented as mean ± SD (n = 10). Difference between groups was considered statistically significant at *P* < 0.05. Note: ^a,b,c^ Statistically significant difference as compared to the corresponding means of naive group (a), EAC group (b), the DOX group (c)

Furthermore, IP inoculation of EAC-challenged mice with ZnONPs/DOX resulted in a significant increase in serum urea level comparing to that in naïve mouse, EAC-challenged mice received PBS and EAC-challenged mice received DOX (245 mg/dl versus 47 mg/dl, 54 mg/dl and 60 mg/dl, respectively) (Table 3). The data in Table 3 indicated that IP inoculation of EAC-challenged mice with ZnONPs, ZnONPs/FA or ZnONPs/DOX/FA has successfully decreased the serum urea level and restored its level to be very close to the naïve mice control (66 mg/dl, 50 mg/dl, and 68 mg/dl) (Table 3). Unfortunately, IP treatment of EAC-challenged mice with DOX, ZnONPs, ZnONPs/DOX, ZnONPs/FA or ZnONPs/DOX/FA resulted in a significant increase in serum creatinine level comparing to that in naïve mice received PBS alone (0.78 mg/dl, 0.79 mg/dl, 0.90 mg/dl, 0.48 mg/dl and 66 mg/dl versus 0.28 mg/dl) (Table 3). IP injection of EAC-challenged mice with ZnONPs/DOX led to a significant increase in the creatinine level (0.90 mg/dl), while their injection with ZnONPs/DOX/FA resulted in a significant decrease in serum creatinine level (0.48 mg/dl) comparing to that in EAC-challenged mice received PBS alone (0.74 mg/dl) (Table 3). Furthermore, EAC-challenged mice injected with ZnONPs/DOX/FA showed a significant decrease in the level of serum creatinine comparing to that in EAC-challenged mice received DOX (0.48 mg/dl versus 0.78 mg/dl) (Table 3).

**Table 3:** Potentials of ZnONP nanocomposites on the kidney function of EAC-challenged mice

Conjugates	Urea (mg/dl)	Creatinine (mg/dl)
Naive	47.67 ± 1.52	0.28 ± 0.04
EAC	54.85 ± 0.63	0.74 ± .05^a^
DOX	60.46 ± 2.04	0.78 ± 0.04^a^
ZnONPs	66.90 ± 6.14	0.79 ± 0.09^a^
ZnONPs/DOX	245.84 ± 21.29^a,b,c^	0.91 ± 0.06^a,b^
ZnONPs/FA	50.90 ± 1.05	0.77 ± 0.3^a^
ZnONPs/DOX/FA	68.15 ± 1.61	0.48 ± 0.03^a,b,c^

Data were represented as mean ± SD (n = 10). Difference between groups was considered statistically significant at *P* < 0.05. Note: ^a,b,c^ Statistically significant difference as compared to the corresponding means of the naive group (a), the EAC group (b), the DOX group (c)

## Discussion

The resistance of patient to the traditional anti-cancer drugs is a major obstacle in cancer chemotherapy and accounts for its failure. As a result of drug non-specificity, multidrug resistance (MDR), and cancer heterogeneity, chemotherapy has a seriously unsatisfactory therapeutic potential against cancer, therefore, there is a consistent need to establish new, efficient, innovative and affordable anti-cancer therapies [3, 46–48]. A nanotechnology-based chemotherapy has become a standard approach in clinical research nowadays, resulting in enhanced therapeutic effectiveness for cancerous tissues and minimal side effects for healthy tissue by targeting cancer cells selectively [15, 36, 49, 50]. ZnONPs had more than 30 times selective cytotoxicity towards cancer cells compared to healthy cells and they could kill cancer cells selectively in vitro and in vivo via inferring the selective localization [15, 19, 21, 49]. The small size and surface properties of ZnONPs enabled them to readily penetrate the blood vessels towards the tumor cells, and to be localized inside these cells specifically, and hence act on them [16, 18, 20].

The main goal of the current search was to investigate the potential of ZnONP nanocomposites as a cancer chemotherapeutic-based drug delivery system and to assess their anti-tumor and anti-inflammatory effectiveness in combination with systemic chemotherapeutic drugs DOX and FA in EAC tumor model both in vitro and in vivo. Overall, our results showed that DOX, ZnONPs, ZnONPs/DOX, ZnONPs/FA and ZnONPs/DOX/FA suppressed the proliferation rate and increased the growth inhibition rate of EAC tumor cells. These results agreed with the findings of Sundraraman [51] who reported that the treatment with ZnONP nanocomposites suppressed the human breast carcinoma proliferation cells and they didn’t show any adverse effect on normal human embryonic kidney cell up to the concentration of 100 µg/ml. Synthesized NPs could arrest the metastasis of breast cancer cell with efficient reduction in tumor volume, lengthening the lifetime of tumor-bearing mice [52].

ZnONP nanocomposites induce apoptosis, cytotoxicity, pro-inflammatory mediator’s production and oxidative stress on human colon carcinoma (LoVo cells) and human hepatocellular adenocarcinoma (HepG_2_) as they decreased mitochondrial activity, loss of normal cell morphology and disturbances in cell cycle distribution [22, 53–56]. Besides, DOX-loaded colloidal NP conferred less cytotoxicity compared to direct treatment with DOX [57]. ZnONP nanocomposites have specific toxicity against cancerous cells by generation of ROS and destruction of mitochondrial membrane potential leading to the activation of caspase cascades followed by apoptosis of cancerous cells [58].

In addition to intercalating base pairs into DNA, DOX inhibits replication, transcription, and topoisomerase II (TOP2) resulting in synthesis inhibition of DNA and RNA. Further, generation of ROS is another mechanism of DOX activity that induces oxidative damage resulting in cleavage or degradation of cancerous cells’ DNA [59, 60]. Apoptosis is one of the most popular ways for anti-cancer medications to generate cancerous cells cytotoxicity [61–63]. Mitochondria, the largest generator of ROS, are considered to be an active molecule in cell death pathways and cause DNA damage [64]. The apoptotic pathway of mitochondria also mediates the genotoxic and cytotoxic potentials of ZnONP nanocomposites, whereas ROS reduced the mitochondrial membrane potential and enhanced the Bax/Bcl2 ratio [65, 66].

Data presented here further demonstrated that combinations of the effective penetration properties of ZnONPs, ZnONPs/DOX, ZnONPs/FA and ZnONPs/DOX/FA resulted in a potent ability to suppress the growth rate of EAC tumor cells in EAC-challenged mice with low cytotoxicity on healthy cells such as splenocytes compared to naïve EAC-challenged mice, possibly due to improved their uptake. This combination significantly augmented cytotoxic and anti-proliferative potentials against EAC tumor cells in EAC-challenged mice increasing the necrosis percentage and apoptosis of EAC tumor cells. Interestingly, ZnONPs alone induced the necrosis and apoptosis rates of EAC tumor cells compared to naïve EAC-challenged mice. These data are in line with the findings of Akhtar et al. [20] and Bai et al. [67] who demonstrated that ZnONP nanocomposites selectively cause significant apoptosis, cytotoxicity and autophagy in cancer cells such as human ovarian cells and gingival cancer cells, which is likely to be mediated by ROS and oxidative stress assembly via p53 pathway and superoxide formation via the mitochondrial intrinsic pathway [68]. Anti-proliferative capability of ZnONP nanocomposites to cancerous cells may be due to the apoptosis induction and destruction of mitochondrial membrane potential leading to the activation of caspase cascades followed by apoptosis of cancerous cells [69].

Our results indicated that the combinational therapy of FA with ZnONPs (ZnONPs/FA) and with ZnONPs/DOX (ZnONPs/DOX/FA) into EAC-challenged mice resulted in increasing the apoptosis rate in EAC tumor cells. FA has a natural affinity towards folate receptor protein, which is over expressed by a number of tumor cells [34]. In addition to its targeted chemo-photothermal therapy synergistic effect, ZnONPs/DOX/FA transported heat and drug conspicuously to cancer cells. Consequently, the ZnONPs/DOX/FA system enhances targeted chemo-photothermal therapy and regulated drug release in a single system [29]. For targeted drug delivery, FA molecules are conjugated to ZnONPs to target folate receptors, which are reported to be overexpressed on many cancer cells [29]. This tumor-targeting compound, FA, allows endocytosis of cancer cells and aggregates ZnONPs by recognizing its homolog, which is commonly expressed on the surface of many cancer cells [30, 33]. Therefore, labeling FA with ZnONPs may be a better medical strategy to target cancer cells as it offers high solubility, long-term diffusion and high biocompatibility in the produced nanomaterial [32, 35].

In the current search, the cytotoxic approach of DOX, ZnONPs, ZnONPs/DOX, ZnONPs/FA and ZnONPs/DOX/FA was experimented against EAC tumor cells, and the results indicated that ZnONPs/DOX, ZnONPs/FA and ZnONPs/DOX/FA were more effective in inhibiting the proliferation rate of EAC tumor cells compared to DOX and ZnONPs and could act as an effective drug delivery system for delivering DOX into EAC tumor cells and improving its chemotherapy effectiveness. The most rationale reason for this improved effectiveness of ZnONPs/DOX and ZnONPs/DOX/FA may be due to high drug loading efficacy, it could markedly increase the intracellular penetration and hence concentration of DOX, thus improving the suppression growth of cancer cells. ZnONPs/DOX revealed effective cytotoxic potential against breast cancer MCF-7 cells and colon cancer HT-29 cells comparing with ZnONPs and DOX alone [36]. The mechanism here may be due to that ZnONPs/DOX, ZnONPs/FA and ZnONPs/DOX/FA caused significant ROS generation, decreased mitochondrial potential and increased caspase-3 activation resulting in induction of mitochondria-mediated apoptosis in tumor cells [36, 37].

Furthermore, the ZnONPs/DOX, ZnONPs/FA and ZnONPs/DOX/FA nanocomposites system enables controlling and targeting drug DOX release in a single system by carrying heat and drug expressly to cancerous cells that increases its uptake and cytotoxicity against cancer cells [22]. The potential of ZnONPs/DOX, ZnONPs/FA, and ZnONPs/DOX/FA in colon carcinoma (HT-29) and breast cancer (MCF-7) cells was reported, confirming their effectiveness in drug delivery to cancerous cells with high therapeutic efficacy and minimum toxicity to healthy cells [26, 70].

Our data revealed that treatment of EAC-challenged mice with ZnONPs, ZnONPs/DOX, ZnONPs/FA and ZnONPs/DOX/FA showed a significant reduction in the levels of pro-inflammatory cytokines IL-6 and TNF-α. A similar effect was reported by Nagajyothi et al. [71], Thatoi et al. [72] and Özcan et al. [73] who reported that NP nanocomposites can influence pro-inflammatory cytokines TNF-α and IL-6 levels by targeting their source cells, modulating their production patterns and interfering with inflammation-related molecules directly through direct interactions with them, such as neutralizing effects, absorption and anergy [74–76]. ZnONP nanocomposites were also shown to induce tumor cells killing capacities of peripheral blood lymphocytes increasing the production of anti-tumor cytokines IFN-γ, IL-2 and TNF-α which further led to the killing of tumor cells and inhibition of tumor growth and enhanced the expression of CD3, CD8, and CD56 [77–80].

Mechanism of ZnONP nanocomposites (ZnONPs, ZnONPs/DOX, ZnONPs/FA or ZnONPs/DOX/FA) as an anti-inflammatory agents may be attributed to their ability to induce the generation of reactive nitrogen species (RNS), suppressing nitric oxide (NO) production, inhibition of expression of inducible nitric oxide synthase (iNOS) enzyme, inhibition of cyclooxygenase-2 (COX-2), inhibition of prostaglandin E2, inhibition of the nuclear factor kappa B (NF-κB) signaling pathway, inhibition of the mast cell degranulation, inhibition of myeloperoxidase and inhibition of the release of pro-inflammatory cytokines IL-6, IFN-γ, TNF-α, IL-17, regulatory cytokine IL-10 and mitogen-activated protein kinases (MAPKS) which were found to be inhibited after blocking internalization of ZnONP nanocomposites through caveolae receptor pathway [71, 81–83].The decrease in the overall level of serum cytokines in ZnONP nanocomposites-treated mice as compared with naïve and naïve EAC-challenged mice may be due to nonspecific immune suppression or even an inequality between the pro-inflammatory and the anti-inflammatory cytokine networks [84].

The findings of the current study demonstrated that treatment of EAC-challenged mice with ZnONPs, ZnONPs/DOX, ZnONPs/FA or ZnONPs/DOX/FA caused marked improvement in the elevated serum levels of ALT, AST, urea and creatinine in EAC-challenged mice that were induced as a result of implantation of EAC cells. This finding is broadly in agreement with Radwan et al. [85] who found that the administration of ZnONP nanocomposites ameliorates the toxicity caused by systemic therapeutic DOX as well as enhances the anti-oxidant defense system in the kidney and liver tissues and exerted a significant decrease in the liver and kidney functions that elevated due to tumor inoculation and systemic therapeutic DOX administration. This is also supported by Nabeel [86] who revealed that ZnONP nanocomposites treatment decreased liver and kidney functions functions enzymes near the normal control. Moreover, ZnONP nanocomposites (ZnONPs, ZnONPs/DOX, ZnONPs/FA and ZnONPs/DOX/FA) exerted cytoprotective effects against ischemic liver injury and kidney injury this renoprotective and hepatoprotective effects might be due to the oxidative stress inhibition, enhancement of cell proliferation, up-regulation of anti-oxidant genes and down-regulation of inflammatory cytokine TNF-α and apoptotic genes caspase-3 and Bax [87]. More studies are required to connect the biological application of the treatment regimen of ZnONP nanocomposites (ZnONPs, ZnONPs/DOX, ZnONPs/FA and ZnONPs/DOX/FA) to therapeutic and diagnostic techniques, however this approach may present a potential regimen for cancer therapy. Our research findings have shown that ZnONPs have an impact on the functioning of the liver and kidney, causing minimal damage to these organs. Although ZnONPs treatment also leads to the generation of ROS in normal cells like hepatic and renal cells, the level of generation is relatively low compared to cancer cells. This is because normal cells initially contain fewer ROS and signaling molecules that can be converted into more reactive species [28]. As a result, the oxidative stress produced may not be enough to cause cell death, leading to a lower cytotoxic response. This could explain the selective cytotoxicity of ZnONPs in proliferating cells, such as cancer cells. By adopting a collaborative approach, it is possible to develop intelligent NPs that specifically target and harm cancer cells without affecting normal cells. This is a realistic goal considering the promising properties of ZnONPs, their inherent selectivity, and their toxicity towards cancer cells. Therefore, ZnONPs can be considered as a valuable tool for the advancement of cancer therapy in the future [28].

## Conclusions

ZnONP nanocomposites may be useful as a cancer chemotherapeutic-based drug delivery system. ZnONP nanocomposites: ZnONPs/DOX, ZnONPs/FA and ZnONPs/DOX/FA regimen may have anti-inflammatory approaches and a great potential to increase anti-tumor effect of conventional chemotherapy, overcoming resistance to cancer systemic chemotherapeutics and reducing their side effects, offering a promising regimen for cancer therapy.

## Data Availability

The data that support the findings of this study are available from the corresponding author upon reasonable request.
